# Research on HIV cure: Mapping the ethics landscape

**DOI:** 10.1371/journal.pmed.1002470

**Published:** 2017-12-08

**Authors:** Karine Dubé, Laurie Sylla, Lynda Dee, Jeff Taylor, David Evans, Carl Dean Bruton, Adam Gilberston, Lisa Gralinski, Brandon Brown, Asheley Skinner, Bryan J. Weiner, Sandra B. Greene, Amy Corneli, Adaora A. Adimora, Joseph D. Tucker, Stuart Rennie

**Affiliations:** 1 University of North Carolina Gillings School of Global Public Health, Chapel Hill, North Carolina, United States of America; 2 defeatHIV Community Advisory Board (CAB), Seattle, Washington, United States of America; 3 amfAR Institute for HIV Cure Research CAB, San Francisco, California, United States of America; 4 Delaney AIDS Research Enterprise (DARE) CAB, San Francisco, California, United States of America; 5 AIDS Action Baltimore, Baltimore, Maryland, United States of America; 6 Collaboratory of AIDS Researchers for Eradication (CARE) CAB, Palm Springs, California, and Chapel Hill, North Carolina, United States of America; 7 Project Inform, San Francisco, California, United States of America; 8 AIDS Clinical Trials Group (ACTG) CAB, Chapel Hill, North Carolina, United States of America; 9 Department of Social Medicine, University of North Carolina at Chapel Hill, Chapel Hill, North Carolina, United States of America; 10 University of California Riverside, Riverside, California, United States of America; 11 Duke Clinical Research Institute (DCRI), Durham, North Carolina, United States; 12 Department of Global Health, University of Washington, Seattle, Washington, United States of America; 13 UNC Institute of Global Health and Infectious Diseases (IGHID), Chapel Hill, North Carolina, United States of America; 14 UNC Project China, Guangzhou, China; 15 UNC Center for Bioethics, Chapel Hill, North Carolina, United States of America

## Abstract

In an essay, Karine Dubé and coauthors discuss the ethics of preclinical and clinical studies relevant to achieving an HIV cure.

**Summary points**

- According to current estimates, 36.7 million people are infected with HIV worldwide. Despite large-scale and growing programs to prevent and treat HIV infection, possible approaches to achieve a cure for HIV infection are of strong interest.
- In the development of candidate approaches to achieve an HIV cure, issues of future translation to human study participants, evidence-based practice, clinical care, diverse populations, and populations in low- and middle-income countries should all be considered.
- An HIV cure should be effective, safe, simple, affordable, and scalable.
- Acceptability research is a critical adjunct to ongoing biomedical HIV cure research efforts.
- Anticipating some of the ethical and implementation challenges related to HIV cure strategies is necessary before the availability of effective interventions.
- Ongoing engagement of stakeholders is needed to resolve ethical, logistical, social, cultural, policy, regulatory, and implementation challenges at all stages of the HIV cure research development process.

There are approximately 36.7 million people living with HIV (PLWHIV) worldwide, and around 1.1 million people died of HIV/AIDS-related complications in 2015 [1]. Global scale-up of HIV antiretroviral therapy (ART) contributed to a 48% reduction in AIDS-related mortality between 2005 and 2016 [2]. Nonetheless, ART does not remove replicative HIV from the body and is not a cure [3]. Without a cure, HIV will remain a chronic infection with the potential to cause and spread lethal disease. Until the Berlin patient’s HIV cure in 2008 [4], a cure for HIV remained inconceivable. The Berlin patient’s cure revived interest in identifying strategies that could lead to long-term remission or elimination of HIV. The Food and Drug Administration (FDA) defines HIV cure research as “any investigation that evaluates: (1) a therapeutic intervention or approach that controls or eliminates HIV infection to the point that no further medical interventions are needed to maintain health; and (2) preliminary scientific concepts that might ultimately lead to such a therapeutic intervention [5].” HIV cure studies occur at various stages of the translational research continuum, from basic scientific discoveries in a laboratory to clinical applications with human participants. As the goal of HIV cure research is to identify regimens that are “effective, simple, safe, and scalable” [6], it will be important to bridge the gap between current basic cure research and the future implementation of cure regimens in clinical practice. In doing so, we must consider ethical issues related to the translation of discoveries from animal models to human participants and future implementation of curative interventions into real-world clinical settings, an area of limited focus in the ethics literature. This paper builds on the emerging HIV cure research ethics [7] to identify key ethical and implementation issues at each stage of the translational research continuum that are distinctive to HIV cure research. We highlight key potential ethical and implementation issues for HIV cure research developers, regulators, and policy makers.

## Translational research continuum and translational ethics

Khoury and colleagues developed a classification framework for translational research that is divided into 4 categories: T0, preclinical research; T1, translation of discoveries to humans; T2, translation of findings to evidence-based practice; T3, translation of interventions to clinical practice; and T4, transition of interventions to improve population health [8]. The T0–T4 translational research continuum provides a unifying framework bridging the range of HIV cure scientific discoveries, human application, advancement into clinical practice, and work towards global HIV cure. We apply this continuum for our analysis of the ethical and implementation issues related to translational HIV cure research. Kagarise and Sheldon are credited with proposing the concept of “translational ethics,” which aims to help stakeholders “navigate the ethical ramifications of technological and scientific advances [9].” Translational HIV cure research will require not only the transfer of discoveries into interventions to improve the lives of PLWHIV but also the effective and responsible translation of interventions into clinical practice and public health [10].

## T0: Preclinical HIV cure research

Most current HIV cure experiments occur in the preclinical (T0) research stage and involve genes, mechanistic pathways, cell lines, and animal models [11]. Significant advancements in HIV treatment, together with increased understanding of animal models for HIV research, have enabled the development of animal models to study HIV persistence and how HIV remains latent inside cells [12]. Nonhuman primates, including rhesus and pigtail macaques, and humanized mice that mimic human immune functions provide useful models to answer scientific questions that inform HIV cure research [12]. Animal models can also allow for control of key variables such as virus dose, route of infection, and host genetics, all of which contribute to infection outcomes. The design of animal model experiments should be scientifically sound to augment the chance that they will be able to predict intervention effectiveness in human studies [13]. This includes ensuring proper sample sizes, applying randomization and blinding, and defining inclusion/exclusion criteria with animal models [13]. There also need to be basic evidentiary thresholds for animal models before interventions are applied to humans [14]. HIV cure scientists must further pay attention to animal research ethics issues, in the context of animals’ intrinsic value and moral rights and independent of their utility in preclinical HIV cure research [15]. The basic tenet of ethical research using animals involves 3 Rs: “*reduce* the number of animals used to the minimum necessary for meaningful results, *refine* procedures to minimize pain and other burdens, and *replace* whole-animal experiments with *in vitro* models or experiments with less sentient organisms whenever possible [16].”

The ethical issues described above are not unique to HIV cure research. What is notable, however, is the pace and scale of preclinical HIV cure research at this time in history, given the significant investments from funders in finding a cure for HIV.

## T1: Translating HIV cure discoveries to humans

The T1 level of translation involves transferring preclinical discoveries to first-in-human (FIH) studies involving Investigational New Drug (IND) applications [8]. These include new investigational approaches to curing HIV (such as gene therapy or stem cell transplants), as well as repurposed drugs (e.g., compounds borrowed from oncology) or novel drugs and biologics advanced from animal models. HIV cure studies face many of the ethical dilemmas surrounding other early-phase research, such as oncology [17]. One key difference, however, is that most HIV cure experiments occur in volunteers who are “otherwise healthy” [18]. HIV cure research is usually conducted among individuals on highly effective and safe antiretroviral therapy, many of whom enjoy an almost normal life expectancy [19]. One of the major ethical issues relates specifically to the high risk of investigational interventions and the corresponding low prospect of direct clinical benefit [20]. HIV cure researchers must make all efforts possible to minimize risks while maximizing the social value of cure studies.

The high risks and minimal prospects for personal clinical benefits of HIV cure research further highlight the significance of a comprehensive informed consent process that distinguishes between benefits to society and benefits to individual participants [21]. The informed consent process must clearly state that HIV cure research is experimental [20], the limited prospect for personal clinical benefits, the unique requirements for the research, and the associated risks, as well as the compensation for research-related injury. In terms of risks, the informed consent process should also make clear any distinctions between risks due to the research modality itself or to monitoring procedures and/or those associated with analytical treatment interruptions (ATIs) [7]. Further, an effective HIV cure will likely require a combination of approaches with the potential of compounding clinical risks beyond those associated with current antiretroviral therapy [22], such as psychosocial, legal, social, and economic risks [23].

In alignment with the principle of distributive and representational justice [24], the selection of HIV cure study participants should remain fair and equitable [7]. Participants in HIV cure research should represent the population of those living with HIV. Inclusion of people of color, women, transgender individuals, and underrepresented minorities in sufficient numbers to achieve statistical power to detect clinical differences is important since gender and genetic differences may affect the biology of curing HIV. Excluding certain populations at early stages may limit results of later stages of translational research [25]. To date, these important subgroups have remained underrepresented in research in general and specifically in HIV cure research [26]. Biomedical HIV cure researchers should engage communities to better understand barriers to subgroup enrollment in clinical studies and inform strategies to encourage greater participation of underrepresented groups [27]. Further, efforts should be made to overcome the challenges of implementing HIV cure research in low- and middle-income countries [28], such as limited clinical and laboratory research capacity, shortages of trained staff, restricted financing, and more fragile healthcare systems [29].

Moreover, HIV cure research encompasses several different HIV cure strategies [30]. Each strategy would benefit from the availability of specific criteria to be used to judge ethical permissibility. For example, Shah argues that experimental stem cell transplantations should not be conducted in children because of the relatively high risks [31]. Sugarman provides consideration for when ART interruption following stem cell transplantation would be ethically appropriate [32]. Future work is needed to create ethical guidelines across types and combinations of HIV cure research strategies. Finally, ATIs remain one of the most controversial topics in HIV cure research that combines considerations for medical, research, and public health ethics. Scholars have provided scientific and ethical considerations related to ATIs, including participant selection, informed consent, community engagement, and public perceptions of ATIs [33,34].

## T2: Translation of findings to evidence-based practice

The T2 level of translation refers to late-phase clinical trials, observational studies, and guideline development [8]. There are few Phase II and III HIV cure clinical trials at present [30]. Further, there are limited validated biomarkers to predict the effects of HIV cure research interventions. The field of HIV cure research could benefit from better harmonization of HIV reservoir assessments and biomarkers and endpoints, within and across types of HIV cure research strategies. The field could also benefit from clear criteria warranting ATIs and benchmarks to define remission success following ATIs [35]. Virologic rebound cannot be predicted during ATIs [36]. Thus, studies involving ATIs must include proper informed consent and frequent virological monitoring. Third-party risks, such as secondary transmissions due to unpredictable relapses of viremia, should be minimized. Adequate standard of prevention packages for the sexual partners of study participants, including information on preexposure prophylaxis (PrEP) or other effective preventive methods [34], should be offered, and sufficient privacy protections should be in place.

A key indicator of HIV cure effectiveness will be the identification of objective biological markers for long-term virological suppression or elimination off therapy. We must define the correct timeframe off ART before we can pronounce a participant cured of HIV. Borrowing the concept of remission from the cancer field [37], a 5-year benchmark for remission is considered a cure in cancer and is being considered by some as a standard for a functional HIV cure.

Another topic of interest in the translation of findings to evidence-based practice will be the substantial level of risk monitoring involved in HIV cure studies. Most interventions carry risks above those of standard ART. For example, histone deacetylase inhibitors (HDACs), a class of latency-reversing agents, can be mutagenic, teratogenic, or genotoxic and may raise concerns among participants about long-term risks of cancer and warrant long-term follow-up monitoring of study participants [38,39].

Biomedical HIV cure trialists must ensure the acceptability of interventions to PLWHIV. The best way to do this is to involve them early on in formulating interventions. Attributes of the interventions will be factors in decisions of PLWHIV to participate and contribute to statistical study power in late-phase efficacy trials. It is not premature to begin assessing hypothetical and actual acceptability of HIV cure interventions with the goal of refining promising HIV cure interventions so that they can be acceptable to PLWHIV [40]. Failure to conduct such assessment may lead to the development of HIV cure research approaches that are unappealing to PLWHIV or otherwise impossible to implement. Thus, acceptability research is a critical adjunct to ongoing biomedical HIV cure research efforts [41].

In addition to study participant acceptability, it is also important to ensure the acceptability of the research and proposed interventions within the larger community. By community, we mean “a group of people with diverse characteristics who are linked by social ties, share common perspectives, and engage in joint action in geographical locations or settings [42].” There is a need for robust community engagement efforts as early as possible in the translational HIV cure research continuum for both PLWHIV and those affected by the epidemic.

## T3: Translation to clinical practice

The T3 level of translation refers to the transfer of effective interventions to clinical practice. T3 also includes implementation research and phase IV clinical trials [8]. At the T3 stage of translation, opinion leader approval is paramount. In the HIV cure research field, there remains a dearth of robust social sciences research around perceptions of HIV care providers [43]. If HIV clinicians are not on board with HIV cure interventions, it is unlikely that patients will receive them. For example, HIV care provider buy-in is one of the facilitators for PrEP uptake in some populations, along with cost and other access issues, as well as considerations about effectiveness and side effects [44]. Further, issues of availability (physical access) and affordability (financial access) of the intervention become prominent at this stage. The HIV cure research field should address the economics of equitable distribution of a cure for HIV. The high costs initially associated with hepatitis C cure inform us of the importance of integrating access factors early in HIV cure development. It would be unethical to deny PLWHIV in the United States and abroad access to a future approved HIV cure because of pricing constraints. Legislators, policy makers, insurance companies, and HIV activists should partner in efforts to ensure that any future HIV cure approaches are accessible to PLWHIV without pricing constraints, especially with regards to low- and middle-income countries [28].

The implications for sexual partners of individuals undergoing curative interventions must also be considered. Of particular concern is the potential for infectivity in the case of viremic rebound [34] and the risk of behavioral disinhibition related to the idea of individuals no longer having HIV and considering themselves uninfectious, possibly leading to secondary infections. PLWHIV will also need to weigh their prospects of a lifetime on antiretroviral therapy [45] versus the potential consequences of undertaking HIV curative regimens. The quality of life of individuals who undergo such HIV cure interventions will need to be monitored regularly, in many cases for years to come. Economic risks of cure will also need to be taken into considerations, such as the possible loss of disability insurance or income [23]. A cure for HIV will likely not reverse the cumulative damage from years or decades of viremia and treatment. Thus, a subset of long-term survivors may have ongoing clinical and psychological issues despite HIV remission or elimination. Further, the possibility of reinfection and eligibility for “second” cures will need to be considered, as it may affect resource allocation decisions as well as HIV prevention efforts.

Finally, at the T3 translation level, implementation research can help determine the best way to operationalize a cure in the real world. We define implementation research as “the study of methods to promote the uptake of research findings into routine practice [46].” While implementation research for HIV cure may seem like a far-fetched and speculative agenda, an effective HIV cure will require a coordinated and integrated approach through all stages of research.

## T4: Translation to improve population health

The T4 level of translation refers to the application of effective interventions to improve population health [8]. If an HIV cure strategy proves successful, a comprehensive rollout strategy that accounts for the ability to scale-up the interventions will be necessary. The scalability of an HIV cure is a key topic in translational ethics, as it refers to the absorptive capacity of healthcare settings to adopt the intervention into existing systems [47]. Scalability must be a key factor in assessing the social value of specific HIV cure research strategies. Efforts are underway to make some HIV cure research approaches more scalable, such as “gene therapy in a box” [48]. To ensure implementation feasibility of an HIV cure around the world, challenges of healthcare worker shortages, limited laboratory capacity and access, financing, quality monitoring, public reluctance to accept new technologies, and priority setting for high-cost interventions will also need to be surmounted [29].

In the context of population health interventions, allocation of scarce resources between HIV prevention, treatment, and HIV cure research represents a translational ethics issue. Fair allocation relates to the principles of distributive justice and responsiveness to global health priorities. Approximately 1.8 million new HIV infections [2] occur each year, and HIV testing and prevention remain important, along with universal HIV treatment for PLWHIV [45]. We have a long way to go in bridging gaps in the HIV treatment continuum worldwide [49]. Development of an HIV cure should closely synergize with ongoing efforts to prevent and treat HIV infection and raise the bar for the entire field of HIV control. For example, HIV cure researchers can work with PrEP clinics to identify acutely infected individuals who may be HIV cure study candidates, although early treatment would restrict, but not prevent, establishment of HIV reservoirs [50]. Moodley and colleagues similarly advocated for a symbiotic relationship between HIV prevention, treatment, and cure in resource-limited settings [51].

Table 1 summarizes some of the considerations for translating HIV cure research discoveries from the T0–T4 stages of translation.

**Table 1:** Considerations for translation of HIV cure research discoveries.

Phases of translation	Considerations
**T0**: **Preclinical research** (animal models in HIV cure research)	• The design of animal model experiments should be scientifically sound so that scientists can predict intervention effectiveness in human studies • Basic evidentiary thresholds for animal models are needed before interventions are applied to humans
**T1**: **Translation of HIV cure research discoveries to humans** (first-in-human studies)	• There is a high risk of investigational interventions and a corresponding low prospect of direct clinical benefit to study participants • There should be a robust informed consent process that distinguishes between benefits to society and benefits to individual participants • The selection of HIV cure study participants should remain fair and equitable (i.e., inclusion of underrepresented minorities) • Ethical guidelines should be created across types and combinations of HIV cure research strategies
**T2**: **Translation of findings to evidence-based practice** (late-phase trials, observational studies, and guideline development)	There is a need for the following: • Better harmonization of HIV reservoir assessment, biomarkers, and endpoints within and across types of HIV cure research strategies • Clear criteria warranting analytical treatment interruptions (ATIs) and benchmarks to define remission success following ATIs • Proper informed consent and frequent virological monitoring for procedures involving ATIs • Standard of prevention packages for the sexual partners of HIV cure study participants • Adequate acceptability research and involvement of people living with HIV (PLWHIV) in formulating interventions • Data collection on implementation costs, training requirements, and other critical translational and implementation issues • Robust community engagement efforts as early as possible in the translational research process
**T3**: **Translation to clinical practice** (transfer to clinical practice, implementation research, and phase IV clinical trials)	• Opinion leaders’ approval (e.g., HIV care providers) will be paramount • Legislators, policy makers, insurance companies, and HIV activists should anticipate challenges related to the economics of equitable distribution of a cure for HIV • The quality of life of individuals who undergo HIV cure interventions will need to be carefully monitored • The possibility of reinfection and eligibility for “second” cures will need to be considered, as it may affect resource allocation decisions and HIV prevention efforts
**T4**: **Translation to improve population health** (application of effective interventions to populations)	• Scalability should be a key factor in assessing the social value of specific HIV cure research strategies • There is a need to anticipate implementation feasibility issues for a global HIV cure, including healthcare worker availability, laboratory capacity, financing, and quality issues • There is a need to consider the allocation of scare resources between HIV prevention, treatment, and HIV cure research • Development of an HIV cure should align with and raise the bar for the entire field of HIV control

## Conclusion

Tremendous human, financial, and social capital is being invested in the discovery of an HIV cure. For an HIV cure regimen to prove valuable, it should be effective, safe, simple, affordable, and scalable [29]. It should also be translatable to human study participants, evidence-based practice, clinical care, and diverse populations. Appreciating the inherent translational ethics issues across the entire research continuum is essential, as HIV cure discoveries must eventually translate to real-world implementation. In this paper, we reviewed some of the considerations at each step of the HIV cure translation and implementation continuum; the issues described are not comprehensive. We asserted that an ethics of translation should begin early in the HIV cure discovery effort, before the availability of efficacious interventions. Logistical, social, cultural, and economic issues will affect the implementation of HIV cure research and interventions at the individual, institutional, national, and global levels. Ongoing community and stakeholder engagement efforts will be crucial to foresee, negotiate, and resolve potential ethical and implementation challenges. Innovative translational and implementation research paradigms utilized at all phases of the HIV cure research continuum will permit us to address critical issues that will ultimately help leverage cutting-edge HIV cure research discoveries to benefit PLWHIV around the globe.

## Abbreviations

ART: antiretroviral therapy
ATI: analytical treatment interruption
FDA: Food and Drug Administration
FIH: first-in-human
HDAC: histone deacetylase inhibitor
IND: Investigational New Drug
PLWHIV: people living with HIV
PrEP: preexposure prophylaxis
